# Intraperitoneal implantation of life-long telemetry transmitters in three rehabilitated harbor seal pups

**DOI:** 10.1186/s12917-017-1060-1

**Published:** 2017-05-25

**Authors:** Markus Horning, Martin Haulena, Justin F. Rosenberg, Chad Nordstrom

**Affiliations:** 1 Alaska SeaLife Center, 301 Railway Avenue, Seward, AK 99664 USA; 2 Oregon State University, 2030 SE Marine Science Drive, Newport, OR 97365 USA; 3 Vancouver Aquarium, 845 Avison Way, Vancouver, BC V6G 3E2 Canada

**Keywords:** Life history transmitter, LHX tag, Telemetry, Vital rate, Implant surgery, Pinniped

## Abstract

**Background:**

Pinnipeds, including many phocid species of concern, are inaccessible and difficult to monitor for extended periods using conventional, externally attached telemetry devices that are shed during the annual molt. Archival satellite transmitters were implanted intraperitoneally into three stranded Pacific harbor seal pups (*Phoca vitulina richardii*) that completed rehabilitation, to evaluate the viability of this surgical technique for the deployment of life long telemetry devices in phocids. The life history transmitters record information throughout the life of the host and transmit data to orbiting satellites after extrusion following death.

**Results:**

Surgeries were performed under general anesthesia and a single transmitter was inserted into the ventrocaudal abdominal cavity via a 7–8 cm incision along the ventral midline between the umbilicus and pubic symphysis or preputial opening in each animal. Surgeries lasted from 45 to 51 min, and anesthesic times ranged from 55 to 79 min. All animals recovered well, were released into dry holding pens overnight, and were given access to water the following day. All three animals exhibited an expected inflammatory response, with acute phase responses lasting approximately three to four weeks. All three animals were tracked via externally attached satellite transmitters after release at 58 to 78 days following surgery, and minimum post-release survival was confirmed through continued movement data received over 278 to 289 days.

**Conclusion:**

The initial findings of low morbidity and zero mortality encountered during captive observation and post-release tracking periods support the viability of this surgical technique for the implantation of long-term telemetry devices in phocids.

**Electronic supplementary material:**

The online version of this article (doi:10.1186/s12917-017-1060-1) contains supplementary material, which is available to authorized users.

## Background

The determination of vital rate data essential to the effective management and conservation of many pinniped species is empirically challenging, especially for those phocid species that do not congregate and breed on specific haulouts or rookeries [1–6]. Essential data include seasonal and age-specific survival, causes and locations of mortality, and age at primiparity and pupping rates in females. Survival and reproductive data are also crucial for the assessment of success and impact of stranded animal rehabilitation programs [7].

Every year, thousands of rehabilitated pinnipeds are released back into the wild in North America [8]. However, no published studies have been able to accurately determine post-release survival beyond four months, nor the social and reproductive behavior, reproductive success, or potential effects on wild populations. The few extant studies have relied on external and internal VHF radio-transmitters, external Argos satellite transmitters, flipper tags, hot-iron branding, color markings and photo-identification. Gaydos and collaborators [9] compared the movement behavior of ten rehabilitated harbor seal pups to ten wild pups. Rehabilitated pups were tracked for a mean of 76.5 days and wild pups for a mean of 132.7 days. No survival rate analysis was conducted. Lander and Gulland [10] tracked three rehabilitated Steller sea lions for 1–4 months, and no other analyses were reported. Lander and collaborators [11] compared the post-release movement and dive behavior between 29 rehabilitated harbor seal pups to and 24 wild pups using VHF transmitters. Animals were tracked for varying periods in different years, up to 179 days. Survival rates were compared up to 14 weeks after release, and were found lower in rehabilitated than wild animals. Morrison and collaborators [12] tracked six rehabilitated harbor seal pups after release using Argos satellite transmitters, and five wild pups for an average of 122 days (rehabilitated) and 150 days (wild). Norris and collaborators [13] used satellite-relayed GPS trackers on six rehabilitated and three wild Hawaiian monk seals for average durations of 91 days (rehabilitated) and 230 days (controls). Vincent and collaborators [14] marked 92 rehabilitated juvenile grey seals via flipper tags, color markings, photo identification satellite tags, or head tags. The photo identification approach allowed the longest periods of resight observation up to five years. Satellite tracking of four of these seals covered periods up to 80 days. 52% of the seals released were never resighted. None of these studies were able to accurately estimate age-specific survival beyond four months after release. The short tracking durations, wide pup dispersal and low visual observation rates also did not allow the determination of social or reproductive behavior.

In marine vertebrates, recent advances in telemetry technology have revolutionized our ability to monitor behavior and movement of individual animals [15]. However, direct telemetric measures of survival and reproduction have been labeled as ‘empirically intractable’ and remain largely elusive [16]. In wild pinnipeds, social behavior, reproductive rates and survival have been accurately documented primarily via mark re-sight approaches using hot-iron branding or natural markings, and often in conjunction with remote video observation systems [17]. These efforts require a large investment in marking and consistent, multi-year re-sight efforts, or the installation, maintenance and operation of remote imaging systems [17, 18]. Such systems have only been used in comparably accessible locations, and on otariid species with a high degree of site fidelity. Such systems are not practicable for phocid species such as harbor seals [19–22]. Reproductive rates for otariids have also been estimated from aerial survey photographic counts of pups in relation to non-pups. Such age ratios can be used to predict population trajectories, or to estimate vital rates such as natality or juvenile survival to inform management decisions for exploited or threatened terrestrial or marine mammals. However, the reliability of such indicators to accurately reflect the underlying dynamics of a population has been criticized [23] and has not been evaluated for marine mammals, and these approaches cannot provide reproductive data on phocids.

Telemetry technology has been tested in a few cases to study survival rates in wild pinnipeds. Externally attached transmitters are not an option due to limited transmitter retention resulting from the annual molt [1, 6, 24]. Subcutaneously implanted transmitters have been used in two studies of harbor seals. Blundell and collaborators [24] used subcutaneous VHF transmitter implants with a battery life of at least three years in 277 harbor seals in Alaska. Transmitters were locally tracked primarily via shore-based VHF receiving data loggers and satellite-linked relay transmitters. These efforts were supplemented via small aircraft VHF scanning flights in the region. As a result of a number of trans-cutaneous tag extrusions observed, combined with tag failures and technical difficulties experienced in tracking the implanted transmitters, the authors concluded that this type of telemetry study using sub-cutaneous VHF transmitters was not suited for the determination of survival rates via electronic mark-recapture studies in harbor seals. Manugian and collaborators [25, 26] used the same type of subcutaneous VHF transmitters implanted into 32 wild harbor seals in California to estimate survival rates. Re-sight effort was conducted via spatially constrained small aircraft VHF scanning survey. However, emigration and possible effects of implants on survival were not evaluated, and tag failure as well as tag loss by extrusion – while observed – were not accounted for. Thus, the reported local apparent survival rates are likely underestimating actual survival by an unknown amount. Prevailing electronic telemetry approaches also do not provide data on birth events in females.

To overcome these constraints and provide vital rate data from pinnipeds in remote, difficult to observe regions, we specifically developed the Life History Transmitter (LHX tag, [27]) in collaboration with Wildlife Computers Inc. (Redmond, WA, USA). LHX tags are intraperitoneally implanted under general gas anesthesia using aseptic surgical procedures [28]. LHX tags record sensor data throughout the life of their host, and have a projected life span of at least 10 years. Summary data are transmitted post-mortem via the Argos satellite data system that provides global coverage with multiple daily uplink opportunities (CLS America, Ltd) only after the tags are liberated from the decomposing, dismembered, or digested carcass [29]. Thus, LHX tags provide the equivalent of end-of-life, known-fate data with spatially and temporally unrestricted data recovery, for at least 10 years. The recommended experimental design using two implants per animal allows the estimation of tag failure rates and data return probabilities. LHX tags store and process data from multiple sensors, including temperature and light. First generation LHX1 tags also incorporated a proprietary sensor to categorize surrounding medium into tissue, water or air [27]. Second generation LHX2 tags (see below) no longer include this sensor and instead incorporate a tri-axial accelerometer sensors used to detect motion and activity. Mortality is determined when temperature falls below a preset threshold. Sensor data is evaluated via a heuristic algorithm to determine state of the host animal, and transmissions are only initiated when tag extrusion has been determined as likely [27]. Two types of data are then transmitted: 48 h of original temperature, light and activity data spanning the detected mortality event, and the parameters of a logistic fit to temperature curves used to detect parturition events, as well as time stamps of detected events. Parturition events are inferred from body core temperature patterns via a modified method first proposed by T. Tinker, as described in [30]. From these transmitted data, exact dates and times of mortality can be obtained, as well as locations of tag emergence via the positioning information provided by the Argos system. From a detailed analysis of transmitted data, inferences may be made on the causa mortis [29, 31]. In some instances of predation resulting in immediate liberation of tags from dismembered hosts, inferences on the nature of predators involved in specific mortality events could be made [31]. If however emergence of tags is delayed and tags cool within an intact carcass, body mass of the deceased animal can be estimated from algor mortis models we previously parameterized for pinnipeds [29]. LHX tags have been used successfully to determine survival and predation rates in juvenile Steller sea lions in the Gulf of Alaska [29, 31, 32].

First generation LHX1 tags were comparably large with a diameter of 42 mm, length of 127 mm, and a mass of 117 g. This limited applications to large animals such as California sea lions (*Zalophus californianus*) and Steller sea lions (*Eumetopias jubatus*). From 2004 through 2014 single or dual LHX tags were implanted into four California sea lions (single tags: 66 and 118 kg, dual tags: 140 and 195 kg) and 45 Steller sea lions (single tags: 109 and 119 kg, dual tags: range 89 to 190 kg, mean 123.6 kg +/− 26 kg S.D.).

**Fig. 1:**
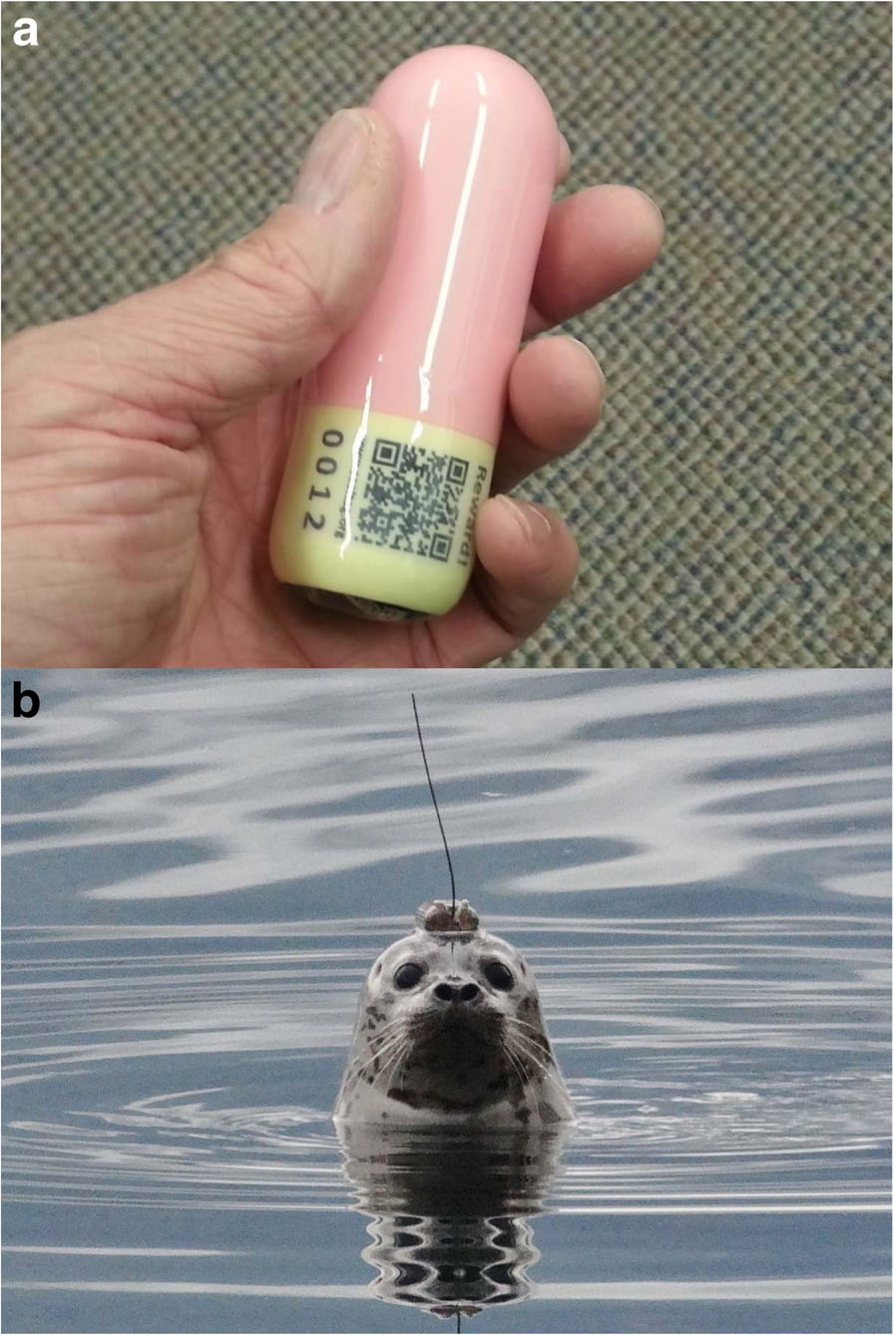
**a** A 2nd generation implantable Argos-compatible satellite-linked life history transmitter (LHX2 tag, Wildlife Computers Inc., Redmond, WA). Exterior dimensions are 97 mm length by 33 mm diameter. The tag mass is 54 g, buoyancy is 0.26 N. The device is coated in Epo-Tek 302-3 M resin, a material certified under the USP Class 6 standard for biological reactivity. The QR code links to information on the tag, project and rewards for return (Photo © Markus Horning). **b** A location-only satellite linked transmitter (SPOT5 tag, Wildlife Computers Inc.) glued onto the head fur via 5-min epoxy, on the just released animal Pv14–123. Exterior dimensions: 70 mm length, 41 mm width, 23 mm height. Tag mass: 72 g (Photo © Sheanna Steingass)

In 2014 we completed the development of 2nd generation LHX2 tags that are smaller to allow for applications in smaller species (Fig. 1). Here we report on the surgery, post-operative monitoring, and post-release tracking of single LHX2 tags implanted into three harbor seal pups that completed rehabilitation, at the Vancouver Aquarium’s Marine Mammal Rescue Centre in 2014. These are the first intraperitoneal telemetry implants in a phocid seal.

## Methods

### Study area

This study was conducted in British Columbia, Canada, between September 2014 and September 2015. Young of the year Pacific harbor seals (*Phoca vitulina richardii*) were implanted at the Vancouver Aquarium Marine Mammal Rescue Centre and were released at Porteau Cove north of Vancouver on November 22, 2014. Seals were subsequently tracked using conventional satellite telemetry for 10 months as they made use of the waters in Howe Sound and the northern portion of the Salish Sea.

### Telemetry transmitters

The 2nd generation Life History Transmitters used in this study have a cylindrical shape with hemispherical ends, with a length of 97 mm, a diameter of 33 mm and a mass of 54 g. Tags are positively buoyant in saltwater (buoyancy 0.26 N). The tags have a pressure rating of >2000 m, and are individually tested to 1000 m. The tags are completely coated in Epo-Tek® 302-3 M medical grade epoxy (Epoxy Technology, Billerica, MA, USA) certified to USP Class VI standards for biological reactivity with implantation in non-cured and polymerized states. Epo-Tek 302-3 M has the lowest moisture absorption amongst Class 6 certified resins, and has exceptionally high resistance to solvents, acids and bases, as well as long-term stability. This material prevents adhesion to connective tissue or omentum. Tags were sterilized in ethylene oxide gas (Anprolene®, Andersen Products Inc., Haw River, NC, USA). The LHX2 transmitters were programmed to transmit abdominal temperatures for postoperative monitoring, every other hour for periods of 12 days following implantation, to a nearby handheld UHF receiver.

For post-release tracking, location only SPOT5 type satellite transmitters (model 287c, Wildlife Computers, WA, USA) were employed using the Argos satellite system for remote data relay. SPOT5 tags measured 70 mm long, 41 mm wide, and 23 mm high and weighed 72 g in air. SPOT5 transmitters were not duty cycled (e.g., they were programmed to transmit whenever possible) but outgoing transmissions were delayed until receiving satellites were predicted to be overhead and transmissions were slowed when seals were hauled-out on land. Animal positions were estimated by Argos CLS America (Maryland, USA) by computing the Doppler shift obtained when satellites passed over transmitting SPOT5 tags and employed a Kalman filter algorithm. Latitudes, longitudes, and quality scores for each derived location were downloaded from the Argos web portal at regular intervals during the tracking period and continued until all tags ceased transmitting. Quality scores reflected the number of satellites in range and the number of successive connections between the transmitting tags and satellites during a given pass with stated accuracies ranging from 250 m to several km (Argos User Manual v. 1.6.6, 2016). Given that the devices were not expected to be recovered and the goal was to maximize deployment longevity, no other parameters were logged or transmitted in order to extend battery capacity for as long as possible.

Tracks for each seal were reconstructed using the trip package [33] and R statistical software [34]. The straight-line distance between successive positions was calculated after outliers were removed from each seal track using a course speed filter set at 5 m per sec (twice the maximum expected speed of 2.5 m per sec for harbor seals) and using locations scored as class ‘B’ or higher. Given the error associated with Argos locations and the inherent gaps in the data record from tags transmitting from diving animals, the distance values reported here should be considered minimum estimates and are solely intended to describe the general movements of the seals in the year following surgery.

### Subjects, anesthesia, and radiographs

Three female harbor seal pups were selected in 2014 that had completed rehabilitation and were deemed releasable based on standard rehabilitation program criteria [35, 36]. Data from a fourth animal (Pv14–099) that did not receive an implant, but was released and tracked at the same time as the three subjects are included here. All four animals were originally admitted as neonates in poor body condition. One animal (Pv14–073) additionally had multiple purulent wounds on both hind flippers, and one (Pv14–062) exhibited monocytosis likely due to chronic inflammation.

Surgeries were performed in a clean, heated shed adjacent to animal holding areas. Starting on the day before surgery and for the following 5 days, subjects were administered a single daily oral dose of the non-steroidal anti-inflammatory drug carprofen (25 mg). Starting on the day after surgery and for the next three days, subjects were orally administered the opioid tramadol (25 mg) twice daily. Subjects were fasted on surgery days, were fed 50% of their normal diet on the day following surgery, and their normal diet was resumed thereafter. Animals were restricted from water access immediately after surgery until the next afternoon, for approximately 18 to 24 h.

Animals were mildly sedated with 0.1 mg/kg midazolam and 0.1 mg/kg butorphanol via intravenous injection into the intervertebral extradural vein. Subjects were then masked with 5% isoflurane (AErrane®, Fort Dodge Animal Health, Fort Dodge, CO, USA) in 100% oxygen for induction of anesthesia [37]. Animals were intubated with a 6.0 mm diameter cuffed endotracheal tube and anesthesia maintained with 1% to 3% isoflurane in 100% oxygen delivered via a semi-closed, partially rebreathing circuit. Depth of anesthesia was assessed based on respiratory rate and tidal volume, response to stimuli, palpebral reflex, capillary refill time, and jaw and muscle tone. Respiratory rate, heart rate, oxygen saturation of hemoglobin (SpO_2_), end-tidal carbon dioxide (EtCO_2_), and deep rectal body temperature were monitored during anesthetic procedures. Animals were provided with mechanically assisted ventilation through a volume regulated ventilator (Model 2000, Hallowell EMC, Pittsfield, MA, USA) at a rate of six to ten breaths per minute and a volume of approximately 15–20 ml/kg/breath if prolonged apnea occurred. A local lidocaine block (5 ml of 5 mg/ml) was performed around the surgical site post-induction. After surgery, isoflurane was discontinued and animals were maintained on 100% oxygen via endotracheal tube during initial recovery. Two view abdominal radiographs were taken for two seals (Pv14–062 and Pv14–073) during recovery. Animals were extubated once jaw tone and the swallowing reflex had returned and they were able to hold their heads up. Recovery occurred within 1 h for all animals, and they were then allowed to recover overnight in dry pens.

### Surgeries

Subjects were positioned in dorsal recumbency and the area from umbilicus to pubis was shaved and prepared with povidone iodine scrub / 70% isopropyl alcohol / povidone iodine solution. A sterile, fenestrated drape was placed over the surgical site and secured with towel clamps. A longitudinal skin incision of approximately 7 cm was made along the ventral midline, caudal to the umbilicus, with a No.10 scalpel. A combination of sharp and blunt dissection through blubber and subcutaneous tissues was utilized to expose the linea alba. The linea alba was then lifted with forceps and incised via stab incision and sharply dissected to expose the peritoneal cavity while avoiding the abdominal contents, to a length sufficient to insert the transmitter (approximately 5 cm). The peritoneum was visualized, incised, and manually expanded. A single, sterilized LHX transmitter was activated, removed from the sterilization peel pouch, rinsed in sterile saline, and inserted into the caudal abdomen through the previously described incision. Bleeding was controlled when necessary with hemostatic forceps and ligatures of 2–0 PDS II absorbable monofilament suture (Ethicon®, Inc., Somerville, NJ, USA). The surgical incision was closed in multiple layers using PDS II sutures. A simple interrupted cruciate pattern with 3–0 PDS II was utilized to close the body wall, ensuring that the external rectus sheath was incorporated into every bite. The thick blubber layer was closed with a simple continuous pattern using 3–0 PDS II. A simple continuous intradermal suture pattern with 3–0 PDS II apposed the skin edges. On two animals only (Pv14–062, Pv14–073), external skin sutures were placed with 3–0 PDS II using interrupted cruciates. The third animal (Pv14–123) did not receive any external skin sutures. Anesthetic times (masking to extubation) and surgical times (incision to completion of sutures) were 66/45, 79/51 and 55/50 min, respectively.

### Postoperative monitoring

Following implantation surgeries, all three seals were closely monitored for 59 to 79 days before release (Table 1). Animals received periodic health screenings and two abdominal view radiographs prior to release to confirm implant positioning. Blood samples were analyzed by a commercial laboratory (IDEXX Laboratories, Delta, B.C., Canada) for hematology and differentials, biochemistry, as well as fibrinogen and haptoglobin concentrations. One day prior to release, animals were sedated with butorphanol and midazolam (each 0.15 mg/kg IV), induced and maintained via mask with isoflurane as described above, and the external SPOT5 satellite transmitters were glued to the head fur using 5-min epoxy (Devcon®, MA, USA).

**Table 1:** Identifiers, dates and body mass of three implanted and one non-implanted female harbor seal pups through rehabilitation and release

Identifier	Admit date	Admit mass (kg)	Admit condition	Surgery date	Surgery mass (kg)	Release date	Release mass (kg)	Days tracked post release
Pv14–073	07/25/14	7.45	poor body condition, wounds	09/02/14	13.00	11/20/14	35.60	278
Pv14–062	07/21/14	7.5	poor body condition, monocyt.	09/02/14	12.45	11/20/14	30.70	289
Pv14–123	08/19/14	8.8	poor body condition	09/22/14	14.30	11/20/14	35.75	284
Pv14–099	08/07/14	5.7	poor body condition, wounds	N/A	N/A	11/20/14	33.65	272

The three implanted seals, and one additional seal (Pv14–099) not carrying an LHX transmitter but carrying a SPOT5 external tag were transported via truck to the release location in Porteau Cove approximately 35 km north of Vancouver in portable kennel cages, and released simultaneously.

### Reference hematology data and descriptive statistics

Reference values for all blood analytes were obtained from the pre-release health examination blood screening records of rehabilitated female harbor seal pups that were released from the Vancouver Aquarium Marine Mammal Rescue Centre in 2014 (*n* = 34) and 2015 (*n* = 28). However, fibrinogen and haptoglobin are not a standard assay on the pre-release exam, and no reference data for harbor seal pups is available. To test for significant effects of surgeries, peak and minimum values for all three experimental animals (the highest and lowest post-surgical value for each individual animal) were compared to the reference values using Student’s two-sample t-test in Systat (Version 10.1). All tests were two-tailed, except leukocytes (1-tailed). Test results are reported as the two-sample t-value_(degrees freedom)_, and *p*-value for alpha = 0.05. Since harbor seal pups continue physiological maturation through the first three months of life (see [38–40]), peaks and minima from weeks one through four after surgery were compared to reference values from pups released prior to three months of age, and peaks and minima for weeks five through 11 after surgery were separately compared to reference values from pups released after three months of age.

## Results

### Body mass, temperatures and general observations

**Fig. 2:**
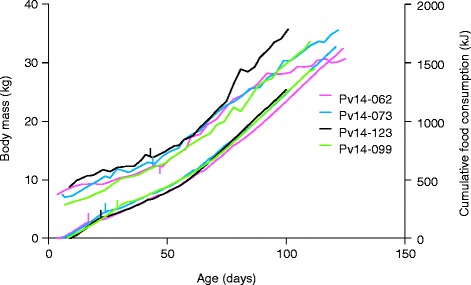
Body mass and cumulative food consumption of four rehabilitated harbor seal pups from admission to release. The transition from formula to whole fish is marked by vertical bars for all four pups on the lower lines showing food consumption. The implant surgeries for three of four subjects are marked by vertical bars on the upper lines showing body mass

All three neonates gained body mass at an average rate ranging from 0.19 to 0.29 kg/d between intake and release. Mass gain rates from intake to surgery were 0.11, 0.15 and 0.16 kg/d. Surgeries occurred at 164, 173 and 160% of intake mass, and release at 409, 478 and 406% of intake mass, respectively (Table 1, Fig. 2). All three subjects briefly lost mass during the pre-surgery and surgery days as a result of restricted diet, and gained mass during the week following surgery at 0.28, 0.24 and 0.20 kg/d.

**Fig. 3:**
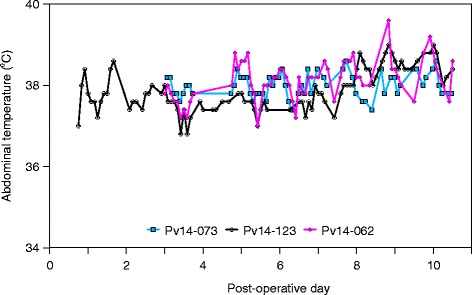
Abdominal temperatures transmitted from the telemetry transmitters intraperitoneally implanted into three rehabilitated harbor seal pups, and received by a nearby telemetry receiver, for up to 10 days after surgery. Due to technical complications, no data were recorded from two of the three animals for the first two days

As a result of technical complications, no transmitted temperatures were recorded from the first two animals for the first three post-operative days. Recorded post-operative temperatures ranged from 36.8 °C to 39.6 °C, and averaged 37.99, 37.91 and 38.11 °C, respectively (Fig. 3).

**Fig. 4:**
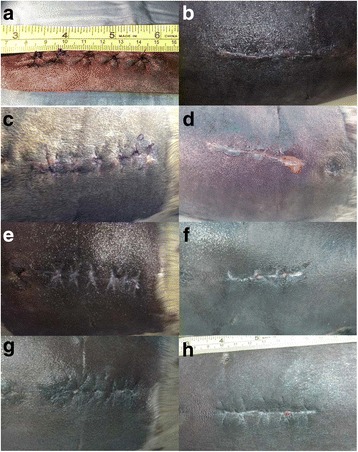
Photos of surgical incisions in three harbor seal pups at varying intervals from surgery to release. **a** Pv14–062 immediately after surgery. External, interrupted cruciate sutures are visible. **b** Pv14–123 on day 4 after surgery. This animal did not receive external skin sutures. **c** Pv14–062 on day 6 after surgery, and before the external sutures were removed. **d** Pv14–123 on day 14 after surgery. There is about a 10 mm section of the incision where the outermost skin layer is separated and not fully apposed after removal of external sutures. **e** Pv14–062 on day 24 after surgery. **f** Pv14–123 on day 28 after surgery. **g** Pv14–062 on day 48 after surgery. **h** Pv14–073 on day 78, prior to release

Incisions measured 75, 78 and 70 mm in length immediately after surgery, and 65, 65 and 60 mm prior to release. Fig. 4 illustrates the progression of incision healing across the three subjects, from surgery to 78 days after surgery just prior to release. One of the two animals with external skin sutures (Pv14–062) exhibited minor swelling and minor purulent discharge from the incision by the end of the first postoperative week (Fig. 4c). Skin sutures were removed 9 days after surgery. The incision was well apposed with no visible evidence of infection, with some granulation apparent by day 9 and good granulation by the end of the second week, and in most cases no visible dehiscence of the skin layer. The incision appeared healed by the 6th week (Fig. 4e, g). The second animal with skin sutures (Pv14–073) exhibited pressure necrosis from these sutures with some purulent discharge by the end of the first week, when skin sutures were removed, and with more discharge observed by day 9. On day 18, the incision was moderately well apposed with a few areas of dehiscence no more than 3 mm at the widest. By 2 weeks, granulation was apparent with some areas of dehiscence (approx. 10 mm) and some discharge. By 24 days, granulation was progressing, but small amounts of discharge persisted through day 46. The incision was mostly healed by day 48 with only 2 small areas remaining to fully close (1–2 mm diameter), and was fully healed by release (Fig. 4h). The animal without skin sutures (Pv14–123) exhibited minor swelling after 1 week, and some discharge on the 2nd week. By the end of the second week small amounts of discharge continued, and a small amount of dehiscence (2–3 mm) was observed (Fig. 4d). By 4 weeks healing had progressed nicely with good granulation, only very minor discharge and a small amount of dehiscence (1–3 mm) on the caudal aspect of the incision (Fig. 4f). This persisted through the release exam on day 58 after surgery, but did not preclude the animal from release.

**Fig. 5:**
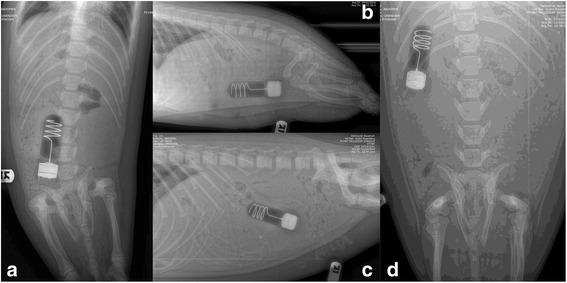
Abdominal radiographs of female harbor seal pup Pv14–062. Immediately after surgery (**a**: dorsoventral, **b**: lateral view) the LHX tag (98 mm length × 33 mm diameter) has a relative length of about 4 vertebrae. 78 days after surgery (**c**: lateral, **d**: dorsoventral view) the LHX tag has a relative length of about 3 vertebrae

In the immediate post-operative abdominal radiographs collected from two animals, the implanted transmitters are positioned just rostral of the pelvis and have a relative length of about 4 vertebrae (Fig. 5a, b). In pre-release radiographs of all three animals the transmitters have moved towards the diaphragm, and have a relative length of about 3 vertebrae (Fig. 5c, d).

### Hematology and clinical chemistry

All data from analyses of blood samples are available in Additional file 1. CBC and differentials were unremarkable on surgery days. Clinical chemistry markers were considered normal for age and diet. Surgeries were completed without complications, and the animals recovered from anesthesia as expected. On the days following surgery, all animals exhibited appetite and consumed all food offered (50% of normal allocation on the day after surgery, 100% thereafter).

**Fig. 6:**
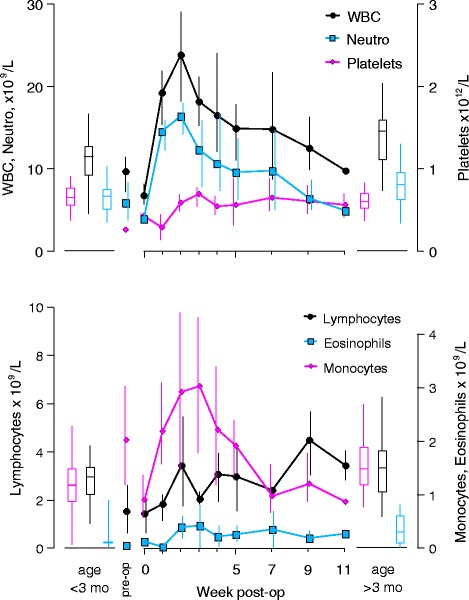
Inflammatory response to implant surgery in three female harbor seal pups. White cell counts (WBC), neutrophils and platelets (upper panel), as well as lymphocytes, monocytes and eosinophils (lower panel) from surgery at approximately 6 weeks of age through release 11 weeks after surgery. Samples were collected at surgery, and at weeks 1, 2, 3, 4, 5, 7, 9 and 11. Pre-surgery values were averaged from available samples collected approximately 4 weeks before surgery. Data points are averages from available samples, and vertical lines connect highest and lowest sample at each week. Reference values for female harbor seal pups under three months of age (whisker boxes on left side of each panel, from *n* = 42 animals), and over three months of age (whisker boxes on right side of each panel, from *n* = 21 animals) were calculated from available data as described under methods

**Fig. 7:**
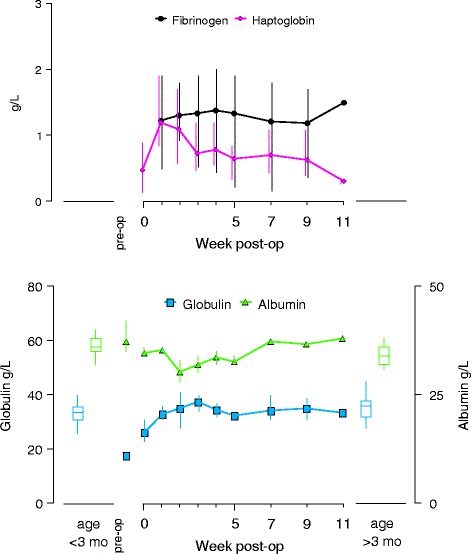
Acute phase response to implant surgery in three female harbor seal pups. Fibrinogen and haptoglobin concentrations (upper panel), as well as globulin and albumin concentrations (lower panel) from surgery at approximately 6 weeks of age through release 11 weeks after surgery. Samples were collected at surgery, and at weeks 1, 2, 3, 4, 5, 7, 9 and 11. No fibrinogen assays were conducted on surgery days. Pre-surgery values were averaged from available samples collected approximately 4 weeks before surgery (lower panel only). Data points are averages from available samples, and vertical lines connect highest and lowest sample at each week. Reference values for female harbor seal pups under three months of age (whisker boxes on left side of each panel, from *n* = 42 animals), and over three months of age (whisker boxes on right side of each panel, from *n* = 21 animals) were calculated from available data as described under methods (lower panel only)

**Fig. 8:**
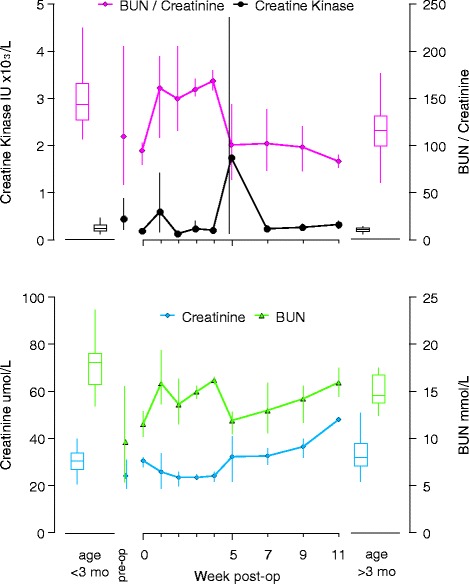
BUN to creatinine ratios and activity of creatine kinase (upper panel), as well as creatinine and BUN concentrations (lower panel) from surgery at approximately six weeks of age through release 11 weeks after surgery. Samples were collected at surgery, and at weeks 1, 2, 3, 4, 5, 7, 9 and 11. Pre-surgery values were averaged from available samples collected approximately four weeks before surgery. Data points are averages from available samples, and vertical lines connect highest and lowest sample at each week. Reference values for female harbor seal pups under three months of age (whisker boxes on left side of each panel, from *n* = 42 animals), and over three months of age (whisker boxes on right side of each panel, from *n* = 21 animals) were calculated from available data as described under methods

During the first four weeks following surgeries, all three animals exhibited inflammatory and acute phase responses (Figs. 6, 7 and 8, data in Additional file 1). Leukocytes (t_(2,41)_ = −5.745, *p* < 0.0001), neutrophils (t_(2,41)_ = −5.9623, *p* < 0.0001) and monocytes (t_(2,41)_ = −7.2105, *p* < 0.0001) exhibited significant peaks compared to reference values (Fig. 5a, b). Eosinophils (t_(2,41)_ = −1.236, *p* > 0.22), lymphocytes (t_(2,41)_ = −1.268, *p* > 0.21) and platelets (t_(2,41)_ = −0.413, *p* > 0.68) tended to increase overall following surgery, but with considerable variance (Fig 6). Fibrinogen and haptoglobin could not be compared to reference values. Fibrinogen exhibited no overall trends when compared to the considerable variance. Haptoglobin levels first increased and then dropped to pre-surgical values by about week three (Fig. 7). Levels of the positive acute phase protein globulin (t_(2,41)_ = −1.9302, *p* = 0.0602) increased relative to reference values without exhibiting a significant peak. The negative acute phase protein albumin exhibited significant post-surgical minima (t_(2,41)_ = −4.24303, *p* = 0.00012) (Fig. 7). Creatinine concentrations exhibited significant minima following surgery (t_(2,41)_ = 3.4708, *p* = 0.0012), and creatinine kinase activity exhibited substantial variation with a single pronounced peak in each animal at distinct times following surgery, resulting in significant peaks compared to the reference pool (t_(2,41)_ = −4.475, *p* < 0.0001). Blood urea nitrogen (BUN) overall increased after surgery, and values for the first four weeks were higher than for weeks five, seven and nine. However, all early post-surgical values were low compared to the reference pool (minima: t_(2,41)_ = 2.6812, *p* = 0.0104). The BUN to creatinine ratio increased sharply after surgery, and dropped by the 5th week, but with considerable variance in subjects and reference pool neither peaks nor minima were significant (Fig. 8).

During weeks five to 11 after surgery, only BUN exhibited significant minima (during weeks five and seven: (t_(2,20)_ = 2.2342, *p* = 0.036), and a single extreme value in creatine kinase in week five resulted in significant peaks compared to controls (t_(2,20)_ = −3.161, *p* = 0.0045). No other analytes exhibited significant peaks or minima compared to the reference values for animals older than three months.

### Post-release telemetry

Animals were tracked for an extended period following release (range = 272 to 289 days). Overall, LHX instrumented seals moved at a mean rate of 27.0 km/d (SE = 1.10, 95% CI = 24.85–29.15) over the course of the deployment (November 2014 to August/September 2015) while Pv14–099 (without the internal transmitter) travelled a nearly identical 26.8 km/d on average over the same time period (SE = 2.70, 95% CI = 21.5–32.1).

Monthly movements were tabulated for each LHX seal to look for any patterns that could indicate difficulty adjusting to life in the wild (e.g., extended at-sea travel, repetitive long-distance movements, or alternatively restricted movements near urban areas such as marinas or docks). No such behaviors were observed other than some long distance movements following the initial release period. However, some individual tendencies were apparent. Pv14–123 routinely travelled less per day (22.9 km, SE = 1.49) than either Pv14–062 (27.9 km, SE = 1.55) or Pv14–073 (29.822.9 km, SE = 1.94) when averaged on a monthly scale. As a result, movements above or below the group’s 95% confidence interval could not be used to generalize inter-animal differences for the three seals. For example, Pv14–123’s movements would be considered less than expected in 8 out of 10 months rather than being within his established range, given this animal’s tendency to move less than the other implanted seals. Instead, a focused search for intra-animal variation and months when multiple seals collectively moved more or less than their established norms was conducted. Specifically, a confidence interval for the monthly average distance traveled per day for each seal was calculated, after removing months with less than 20 days of data (typically the first and last months of the deployment; Table 2). When the average movement in any given month was exceptional for that particular animal the average movement of other implanted seals was considered. Movements in 53% of cases exceeded an individual seal’s confidence interval but no regular patterns were apparent among seals beyond a tendency to travel more in the first two months after leaving the relatively protected waters of Howe Sound. The exception was the month of July where all 3 seals moved in an atypical manner but while two animals moved slightly more than expected, the third moved notably less than its established average. Similarly, no patterns were observed when movements were examined by season with mean rates changing little over time and ranging from 24.9 km/day (summer) to 26.4 km/day (spring).

**Table 2:** Identifiers and summary statistics (mean, standard deviation, standard error, and 95% confidence intervals) for the monthly averaged daily movements (km) of three implanted and one non-implanted harbor seal pups post-release

Identifier	Mean daily km per month	SD	SEM	CI	Lower CI	Upper CI
Pv14–073	29.8	5.83	1.94	3.81	25.96	33.57
Pv14–062	27.9	4.66	1.55	3.04	24.93	30.92
Pv14–123	22.9	4.21	1.49	2.92	19.94	25.77
Pv14–099	26.8	8.11	2.70	5.30	21.51	32.11

All four external satellite tags ceased transmitting within 17 days of each other, indicating that the instruments likely exhausted their batteries or fell off during the annual molt.

Through February 2017, no data returns from the implanted LHX tags were received.

## Discussion

The three subjects were admitted into the Vancouver Aquarium Marine Mammal Rescue Centre as neonates less than one week old. Intake body mass was below the 11.2 kg average reported for wild newborn harbor seal pups in the study region [41]. All three pups completed rehabilitation procedures and were deemed releasable when they were selected for implantation surgery. Growth rates during the rehabilitation period averaged 0.14 kg/d, which is below the rate reported for wild harbor seal pups in the region of 0.39 kg/d [41], but within the range of 0.04 to 0.55 kg/d reported for harbor seal pups in rehabilitation [42]. Surgery occurred at an age of a little over 6 weeks, by which time wild harbor seal pups are typically weaned [41]. At this age harbor seal pups still grow and develop, and continue to undergo physiological maturation and adjustments. Weight gain for the week following surgery averaged 0.24 kg/d, which was also the overall average weight gain from admit to release. This suggests that within the constraints imposed by stranding and rehabilitation, food intake, digestion, assimilation, and the resulting growth rates were not affected by the surgeries or the implanted telemetry devices.

Physiologically, the three pups exhibited the expected immunological and acute phase responses.

White blood cell counts increased one week after surgery, peaked at two weeks and then declined. Levels had declined to within the reference range by week five, and had returned to normal at the time of release at week 11. This appeared largely driven by mature neutrophils (absolute values) and monocytes, which showed an identical pattern. Eosinophils also increased after surgery but with considerable variance between samples and individuals. Platelets increased in all three subjects, but with considerable variance, and remained elevated but within reference ranges throughout the observation period. Lymphocytes increased overall and varied considerably, but remained within reference ranges. The two subjects that received skin sutures exhibited two peak lymphocyte values each above normal ranges, whereas the third subject did not.

The acute phase protein response was qualitatively as expected. The positive response protein haptoglobin increased and peaked at one week post-operative, after which values declined. Pre-operative values were only obtained from two animals. Comparable post-operative values were reached in week three. No pre-operative fibrinogen values were obtained, the post-operative values showed no clear trend within large variance. Pre-operative levels of globulin (a positive response protein) were below reference values, increased to within reference range with a peak at three weeks, and then declined slightly but remained within the reference range. The negative response protein albumin declined only during the 2nd week, after which values gradually increased.

Creatinine concentrations dropped following surgery and some values were below reference ranges for pups younger than three months. Values returned to pre-surgery levels by week five, and further increased up until release. By release, values were highest, but still within the reference range for pups older than three months. The decrease in creatinine could be related to temporarily reduced food intake, reduced activity in the small intestine and pancreas, or less likely due to increased kidney function. All three animals exhibited highly varying levels of creatine kinase (CK), with each showing a pronounced peak at one, three, and five weeks after surgery, respectively. In one animal (Pv14–073) the CK peak at one week preceded a drop in creatinine, and in two animals the peaks coincided or preceded increases in creatinine levels. Increased CK levels may be indicative of damage to tissue with already high normal levels of CK (such as muscles) and were likely secondary to surgery, or possibly from hematomas due to multiple weekly venipunctures. No other analytes from the animals exhibiting these single high CK values at those times were concerning, suggesting that these isolated high values were not clinically relevant. Blood urea nitrogen increased, and the BUN to creatinine ratio increased sharply after surgery, and dropped by the 5th week (Fig. 8). However, BUN values were below the reference range just prior to surgery and then increased to within this range. While an increase in BUN/creatinine could be indicative of kidney injury or gastrointestinal bleeding, or due to the processing of large hematomas, this is unlikely given the overall low values. A possible cause of the generally low BUN values prior to surgery and around week five may be in the combination of temporarily reduced food intake prior to surgery, and possibly reduced exercise after surgery. Generally, BUN, creatinine and CK values appeared to vary considerably but remained broadly within the wide reference ranges and were not deemed related to clinically relevant complications.

Overall, the observed biochemical changes appear more reflective of a response to surgery, tag insertion and inflammation, rather than an immune response. Electrolytes were unremarkable and exhibited age and high protein diet-related changes. The initial, acute response lasted through approximately week four, by which time acute response proteins had largely returned to pre-surgery values. Similarly, the initial inflammatory response peaked between the second and third week, after which some levels normalized. Following this acute response phase, all analytes with a few isolated exceptions did not indicate the persistence of a protracted, chronic response to surgery or implants.

The post-release tracking data confirms that all three pups survived at least their first year post surgery. Despite individual differences in the movement rates among LHX seals, the mean daily and seasonal movement rates were nearly identical to the non-surgical seal from the same cohort that was tracked simultaneously, and were generally within the range observed in rehabilitated harbor seal pups released by the Rescue Centre in 2013 (*Haulena unpub data)*. In contrast, movement rates were much greater than rehabiliated pups and wild pups instrumented further south in the adjacent waters of Puget Sound in Washington State [9]. However, given that transmission duration was also at least twice as long for our deployments further north in the more open areas of the Salish Sea, it’s more likely that differences in movements are more reflective of geography than of a result of the LHX implantation procedure. The extended deployments allowed the opportunity to assess coarse changes in seal movements over nearly a year but few patterns were apparent on a monthly or seasonal basis. Movement rates were highly individualistic which prevented us from describing any trends or potential development in the movements of the rehabilitated pups. Nevertheless, the movements of all seals did slow during the final months of the recorded deployments when compared to their previous months (August or September). Reduced activity is associated with the late summer/early fall molting period but in our case the results were confounded by incomplete monthly records (as the tags were likely shed before recording most of the final month).

The absence of post-mortem transmissions from LHX tags further suggests a high likelihood that all three animals survived through their second year. However, the reliability of these 2nd generation LHX2 tags has not yet been empirically validated in wild animals. Three of nine 2nd generation LHX2 tags deployed in combination with 1st generation tags in 9 weaned female Steller sea lions in 2014 transmitted after their host died, demonstrating their functionality. 1st generation LHX1 tags deployed in 36 weaned, juvenile Steller sea lions in the Gulf of Alaska between 2005 and 2014 had a 87% individual tag probability of uplinking to the Argos satellite system after the host animal had died [31]. If data return probability of 2nd generation tags is comparable, this would indicate a very high probability that all three pups were still alive two years following implantation surgery.

Despite significant recent advances in anesthesia and veterinary surgical techniques, abdominal surgeries in pinnipeds are still considered a rare option for treatment of captive or rehabilitated animals, or for deployment of internal telemetry transmitters [43]. To date, Steller sea lions are the only pinniped species in which intraperitoneal implants have been conducted on a larger scale (45 animals), and where the impact of implants has been studied in detail both before and after release [28, 29, 31, 44–50]. Based on the analysis of blood samples from the initial six sea lions, an acute inflammatory post-operative response was observed, primarily in the form of an increase in haptoglobin concentrations. This response lasted through five weeks, with a return to pre-surgery levels by the 6th week [45, 47]. From this, the authors derived a six week study inclusion criterion for studies based on LHX implants: survival has to be demonstrated to six weeks for data to be included in vital rate studies. Post-operative body core temperatures transmitted from the implants of all 45 sea lions ranged from 36 to 38.2 °C, though in a few instances a response to localized inflammations at the incision led to temperatures as high as 39.4 °C [28, 31]. By comparison, post-operative temperatures in the three harbor seal pups averaged at the upper end of the normal temperature range observed for Steller sea lions. A similar peak temperature of 39.6 was observed. In Steller sea lions, a comparison between a subset of 35 of the implanted animals, and 27 free-ranging control animals revealed no difference in cumulative survival from ages 14 months to 5 years, though due to the small sample sizes the power of the comparison was very low [50].

In phocids, no intraperitoneal implants have been used prior to this study. Four different types of subcutaneous implants have been tested through extended postoperative monitoring in 10 harbor seals [51]. However, no detailed longitudinal health assessments were reported other than CBC and serum biochemistry values that were within normal ranges 1 week after surgery for 9 of the 10 subjects. From visual observations of tissue reaction and wound healing, the authors concluded that one type of wax coated implant led to fewer tissue reactions and was preferable for long-term studies. Another five wild harbor seals received this type of implant and were released without extended postoperative monitoring. As described in the background section, two studies used subcutaneous implants to determine survival rates in wild harbor seals. One study on 32 seals did report apparent survival rates but without correcting for emigration, tag failures and extrusion, or tag effects on survival [25, 26]. The second study on 277 seals concluded that this technique was not suited to the estimation of survival rates via telemetric mark re-sight experiments. Conversely, a much smaller sample size of 45 Steller sea lions implanted with dual LHX tags delivering known fate data via temporally and spatially unrestricted satellite transmissions with a better than 98% post-mortem data return probability (for dual tag deployments), has already led to findings on survival and causes of mortality that could not otherwise be obtained in pinnipeds [29, 31, 32].

## Conclusions

The low post-operative morbidity, expected clinical effects of surgeries and implants, and zero mortality during at least the first year after release illustrate the survivability of implantation surgery and intraperitoneal telemetry transmitters in phocid seals. This in turn suggests LHX tags as a viable technology to study vital rates in phocid seals, and to evaluate the efficacy and impact of rehabilitation programs. Additional studies with a larger sample size, possibly on non-rehabilitated animals, could lead to a greater statistical power on the nature and duration of the acute phase inflammatory response, and may yet reveal other tag effects not apparent here. This may also allow the establishment of a shorter study inclusion criterion than the five to six weeks suggested by these initial results, for vital rate studies in phocids using LHX tag technology. Any additional studies using LHX tags in harbor seals should furthermore continue to monitor for any long-term effects on behavior, reproduction and survival.

## Additional file

Additional file 1: All results from analyses of blood samples in Microsoft Excel workbook format (XLSX 29 kb).

## Abbreviations

BUN: Blood urea nitrogen
CBC: Complete blood count
CK: Creatine kinase
LHX: Life History Transmitter
UHF: ultra high frequency
VHF: very high frequency
